# IMPACT OF GERIATRIC INTERPROFESSIONAL TRAINING ON ADVANCE CARE PLANNING IN GERIATRIC PRIMARY CARE

**DOI:** 10.1093/geroni/igz038.544

**Published:** 2019-11-08

**Authors:** Maureen E Barrientos, Anna Chodos, Alicia Neumann, Yvonne Troya, Pei Chen

**Affiliations:** 1 University of California San Francisco, San Francisco, California, United States; 2 University of California Hastings College of the Law, San Francisco, California, United States

## Abstract

Currently, an important measure of Advance Care Planning (ACP), Advance Health Care Directives (AHCD) documentation rate, is at 33% for older adults in the United States. To address this disparity, geriatric faculty in an academic geriatric primary care practice aimed to train geriatrics fellows and other interprofessional (IP) learners to engage patients in ACP. As part of a Geriatric Workforce Enhancement Program funded by the Health Resources and Services Administration, geriatrics faculty and the Medical Legal Partnership for Seniors based at University of California Hastings College of Law provided ACP training to fellows and IP learners, including social work interns. In practice, the fellows and social work interns collaborated to incorporate ACP into patient visits and follow-up telephone calls. To monitor ACP progress, research staff reviewed patients’ electronic health records and performed descriptive analysis of the data. In 21 months, 4 geriatrics fellows built a panel of 59 patients who on average had 3 office visits and 7 telephone calls per person. Prior to clinic enrollment, 12 (20.3%) patients had preexisting AHCD, and 47 lacked AHCD documentation. After ACP intervention, 42 of 47 patients without AHCD documentation engaged in ACP discussion. Of those who engaged in ACP discussion, 24 completed AHCD, raising AHCD completion rate to 61%, or 36 patients in the panel of 59. ACP is a complex process that benefits from skilled communication among interprofessional providers and patients. Findings underscore the potential advantages of IP training and engaging patients in ACP discussion in an academic primary care setting.

